# Insecticidal and oviposition deterrent effects of essential oils of *Baccharis* spp. and histological assessment against *Drosophila suzukii* (Diptera: Drosophilidae)

**DOI:** 10.1038/s41598-021-83557-7

**Published:** 2021-02-17

**Authors:** Michele Trombin de Souza, Mireli Trombin de Souza, Daniel Bernardi, Douglas José de Melo, Paulo Henrique Gorgatti Zarbin, Maria Aparecida Cassilha Zawadneak

**Affiliations:** 1 Department of Basic Pathology, Federal University of Parana, Mailbox 19031, Curitiba, PR 81531-980 Brazil; 2 Department of Plant Health, Federal University of Pelotas, Mailbox 354, Capão-do-Leão, RS 96010-900 Brazil; 3 Department of Chemistry, Federal University of Parana, Mailbox 19020, Curitiba, PR 81531-990 Brazil

**Keywords:** Natural products, Natural product synthesis, Invasive species

## Abstract

The diverse flora of the Atlantic Forest is fertile ground for discovering new chemical structures with insecticidal activity. The presence of species belonging to the genus *Baccharis* is of particular interest, as these species have shown promise in pest management applications. The objective of this study is to chemically identify the constituents expressed in the leaves of seven species of *Baccharis* (*B. anomala* DC., *B. calvescens* DC., *B. mesoneura* DC., *B. milleflora* DC., *B. oblongifolia* Pers., *B. trimera* (Less) DC. and *B. uncinella* DC.) and to evaluate the toxicological and morphological effects caused by essential oils (EOs) on the larvae and adults of *Drosophila suzukii* (Diptera: Drosophilidae). Chemical analysis using gas chromatography-mass spectrometry (GC–MS) indicated that limonene was the main common constituent in all *Baccharis* species. This constituent in isolation, as well as the EOs of *B. calvescens*, *B. mesoneura*, and *B. oblongifolia*, caused mortality in over 80% of adults of *D. suzukii* at a discriminatory concentration of 80 mg L^−1^ in bioassays of ingestion and topical application. These results are similar to the effect of spinosyn-based synthetic insecticides (spinetoram 75 mg L^−1^) 120 h after exposure. Limonene and EOs from all species had the lowest LC_50_ and LC_90_ values relative to spinosyn and azadirachtin (12 g L^−1^) in both bioassays. However, they showed the same time toxicity over time as spinetoram when applied to adults of *D. suzukii* (LT_50_ ranging from 4.6 to 8.7 h) in a topical application bioassay. In olfactometry tests, 92% of *D. suzukii* females showed repellent behavior when exposed to the EOs and limonene. Likewise, the EOs of *B. calvescens*, *B. mesoneura*, and *B. oblongifolia* significantly reduced the number of eggs in artificial fruits (≅ 7.6 eggs fruit^−1^), differing from the control treatment with water (17.2 eggs fruit^−1^) and acetone (17.6 eggs fruit^−1^). According to histological analyses, the L3 larvae of *D. suzukii* had morphological and physiological alterations and deformations after exposure to treatments containing EOs and limonene, which resulted in high larval, pupal, and adult mortality. In view of the results, *Baccharis* EOs and their isolated constituent, limonene, proved to be promising alternatives for developing bioinsecticides to manage of *D. suzukii*.

## Introduction

The genus *Baccharis* (Asteraceae) comprises 435 species found exclusively in the Americas, with records from the south of Canada to southern South America^1^. In Brazil, 179 species have been described, most of which occur in the southern region of the country^2^. *Baccharis* spp. are distributed throughout the Atlantic Forest biome, a global hotspot of biodiversity that contains more plant species than other Brazilian biomes, with over 19,000 species, of which 7,600 are endemic^3^. Despite the remarkable levels of endemism that make the Atlantic Forest one of the most distinct regions of the Neotropics^2,3^, little is known about the potential genetic resources of aromatic plants present in this biome. Studies have only been carried out to verify the potential for biological control against arthropod pests for 27 species of *Baccharis*^4^.

One important characteristic of *Baccharis* is the presence of secondary metabolites, specifically essential oils (EOs), which have a rich composition of terpenes that includes monoterpenes, sesquiterpenes, diterpenes, and triterpenes^1,4,5^. The EOs of *Baccharis* spp. have been used for centuries as therapeutic agents in traditional medicine due to their spasmolytic, diuretic, anti-inflammatory, antibacterial, and antifungal properties^1,4^. In addition, these EOs have been recognized for their fumigant^1^, larvicidal^6^, toxic and repellent^7^ effects against arthropods. Similarly, certain individual constituents of the oils, such as limonene, can cause the dissociation of lipids present in the cuticle of the exoskeleton of insects, causing dehydration and death^8,9^.

Several EOs have shown promise for agricultural applications, mainly against mites and insects, including *Drosophila suzukii* Matsumura (Diptera: Drosophilidae), a major pest of thin-skinned fruit with a global distribution^10–12^. The serrated ovipositor of *D. suzukii* females allows them to lay their eggs in healthy and ripe fruits, leading to economic damage^13–15^. Meanwhile, developing larvae can cause the fruit to soften and result in rapid decomposition, making the fruit unsellable^13,15^.

The management of *D. suzukii* is challenging due to its wide range of hosts, short biological cycle^16–18^, and wide environmental adaptation^19–21^. Although synthetic spinosyn-based insecticides are available for the control of *D. suzukii*, these products require a preharvest interval of 5 to 14 days^22^. However, frequent applications may be necessary to keep the population level low^23–25^, meaning that there is a risk of pests developing spinosad resistance if producers do not alternate with a different chemical class^26,27^. In addition, the cultivation of small fruits in major producing countries, such as Brazil, is carried out on small properties that use organic or low-residue practices, and where the use of synthetic substances is restricted or prohibited^28^. Thus, EOs can be an alternative for the management of *D. suzukii*^9,13,29^. This is because EOs have multiple modes of action that can reduce or prevent the evolution of resistance^30^. They can also be used in organic production systems due to their high volatility and absence of residues on fruits^9^.

Therefore, this study aims to: (i) characterize and isolate the main common constituents present in the leaves of seven species of *Baccharis (B. anomala, B. calvescens, B. mesoneura, B. milleflora, B. oblongifolia, B. trimera*, and *B. uncinella*); (ii) evaluate the lethal toxicity of the EOs and isolated constituents on adults and larvae of *D. suzukii*; (iii) to assess the repellent effect of dry EO residues on oviposition by *D. suzukii*; and (iv) to analyze the morphological damage caused by EOs to the target organs of *D. suzukii* larvae, such as the brain, fat body, and Malpighian tubules using a histological assessment.

## Results

**Figure 1:**
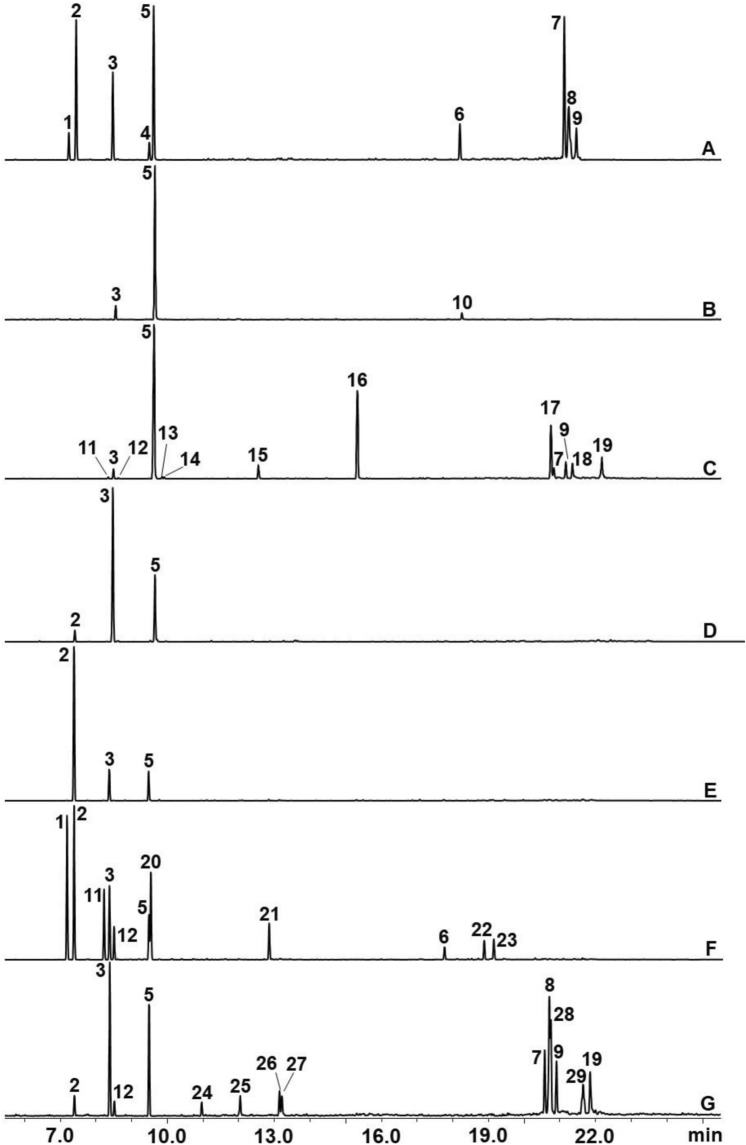
GC/MS chromatogram of essential oil of species de *Baccharis*: (**A**) *B. calvescens*; (**B**) *B. uncinella*; (**C**) *B. trimera*; (**D**) *B. milleflora*; (**E**) *B. mesoneura*; (**F**) *B. oblongifolia*; (**G**) *B. anomala*. Chemical constituents: **(1)** α-thujene; **(2)** α-pinene; **(3)** β-pinene; **(4)** o-cymene; **(5)** limonene; **(6)** caryophyllene; **(7)** spathulenol; **(8)** thujopsan-2-α-ol; **(9)** viridiflorol; **(10)** farnesene; **(11)** sabinene; **(12)** β-myrcene**; (13)** β-phorone; **(14)** β-ocimene; **(15)** carveol; **(16)** carquejyl acetate; **(17)** palustrol; **(18)** Ledol; **(19)** β-eudesmol; **(20)** β-phellandrene; **(21)** terpinen-4-ol; **(22)** germacrene-D; **(23)** bicyclogermacrene; **(24)** linalool; **(25)** trans-pinocarveol; **(26)** α-terpineol; **(27)** myrtenol; **(28)** globulol; **(29)** α-muurolol.

In total, 29 chemical constituents were identified in the EOs from the samples of *Baccharis* spp. (Table 1, Fig. 1). These constituents comprised monoterpene hydrocarbons (which represented 34.9%–100% of EO constituents), oxygenated monoterpenes (5.3–25.1%), sesquiterpene hydrocarbons (3.6–8.0%), and oxygenated sesquiterpenes (29.2–9.8%) (Table 1). Limonene was the main common constituent present in all species (12.5%–88.8%; Table 1). Other chemical constituents with a high relative proportion (%) included α-pinene (15.7%), β-pinene (11.8%), spatulenol (21.3%), and thujopsan-2-α-ol (13.2%) in *B*. *calvescens*; carquejyl acetate (22.0%) and palustrol (13.1%) in *B. trimera*; β-pinene (67.5%) in *B. milleflora*; α-pinene (72.6%) and β-pinene (14.1%) in *B. mesoneura*; α-thujene (20.2%), α-pinene (22.1%), and β-pinene (10.8%) in *B. oblongifolia*; and β-pinene (18.3%), thujopsan-2-α-ol (17.7%), and globulol (10.9%) in *B. anomala* (Table 1, Fig. 1).

**Table 1:** Essential oil composition (%) of samples fresh leaves of *Baccharis* spp.

Constituents	RI^lit^	RI^cal^	% peak area						
			1	2	3	4	5	6	7
α-thujene	924	926	3.4	–	0.3	4.2	–	20.2	–
α-pinene	932	935	15.7	–	–	–	72.6	22.1	2.1
sabinene	973	973	–	–	–	–	–	9.9	–
β-pinene	974	974	11.8	7.6	2.0	67.5	14.1	10.8	18.3
β-myrcene	988	990	–	–	0.2	–	–	4.4	1.5
o-cymene	1022	1024	2.3	–	0.3	–	–	–	–
limonene	1024	1026	20.6	88.8	42.2	28.2	13.3	12.5	13.0
β-phellandrene	1029	1033	–	–	–	–	–	6.6	–
β-phorene	1043	1041	–	–	0.4	–	–	–	–
β-ocimene	1044	1041	–	–	0.3	–	–	–	–
Monoterpene hydrocarbon			53.8	96.4	45.7	99.9	100.0	86.5	34.9
terpinen-4-ol	1181	1177	–	–	–	–	–	5.5	–
α-terpineol	1188	1894	–	–	–	–	–	–	3.0
myrtenol	1194	1197	–	–	–	–	–	–	2.3
linalool	1099	1095	–	–	–	–	–	–	–
trans-pinocarveol	1135	1137	–	–	–	–	–	–	–
carveol	1216	1216	–	–	3.1	–	–	–	–
carquejyl acetate	1298	1299	–	–	22.0	–	–	–	–
Oxygenated monoterpene			0	0	25.1	0	0	5.5	5.3
*(E*)-caryophyllene	1417	1418	4.9	–	–	–	–	1.7	–
β-farnesene	1442	1442	–	3.6	–	–	–	–	–
germacrene D	1484	1485	–	–	–	–	–	3.0	–
bicyclogermacrene	1500	1500	–	–	–	–	–	3.3	–
Sesquiterpene hydrocarbon			4.9	3.6	0	0	0	8.0	0
palustrol	1577	1576	–	–	13.1	–	–	–	–
spathulenol	1577	1576	21.3	–	2.6	–	–	–	8.0
thujopsan-2-α-ol	1587	1590	13.2	–	–	–	–	–	17.7
globulol	1590	1595	–	–	–	–	–	–	10.9
viridiflorol	1592	1592	4.4	–	3.7	–	–	–	7.2
ledol	1602	1602	–	–	3.7	–	–	–	3,7
α-muurolol	1644	1647	–	–	–	–	–	–	6.3
β- eudesmol	1650	1653	–	–	6.1	–	–	–	6.0
Oxygenated sesquiterpene			38.9	0	29.2	0	0	0	59.8
Total chemical composition (%)			97.6	100	100	99.9	100	100	100

*RI*^*lit*^ Literature Retention Index, *RI*^*cal*^ Experimental Retention Index.

^sp^Species: 1 *B. calvescens;* 2 *B. uncinella;* 3 *B. trimera*; 4 *B. milleflora*; 5 *B. mesoneura;* 6 *B. oblongifolia;* and 7 *B. anomala*.

–Constituents not present.

**Figure 2:**
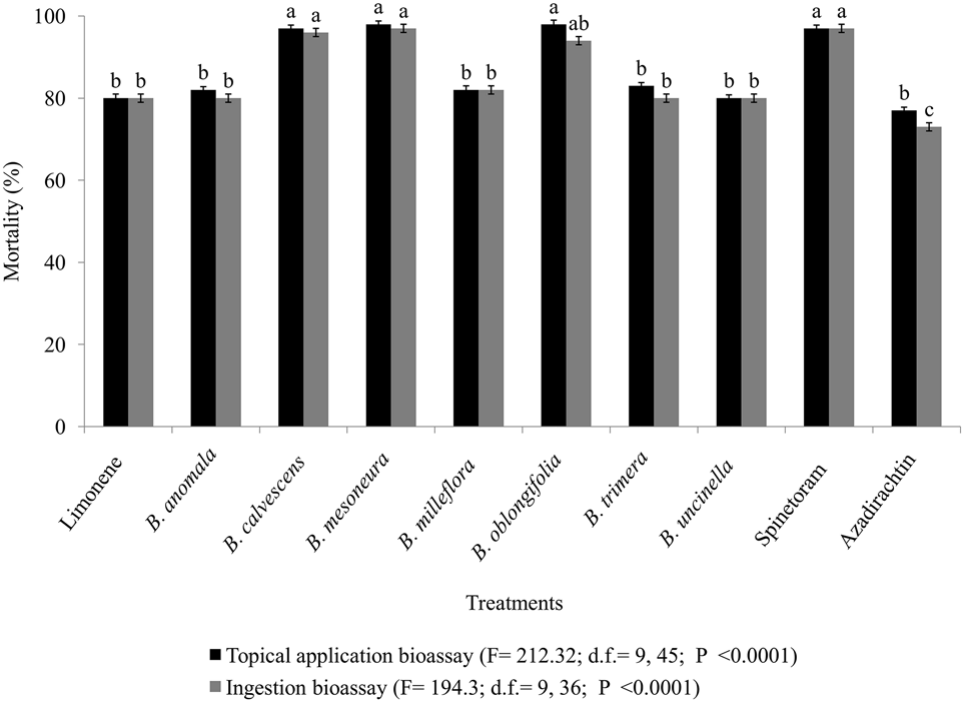
*Drosophila suzukii* mortality when submitted to various treatments in topical application and ingestion bioassays. Means followed by different letters on the columns (within each exposition bioassay) indicate significant differences between treatments (GLM with quasi-binomial distribution followed by post hoc Tukey test, *P* < 0.05).

After 120 h of exposure, discriminatory concentrations of the EOs of *B. calvescens*, *B. mesoneura*, and *B. oblongifolia* and spinosyn (75 mg L^−1^) showed high toxicity, with *D. suzukii* adult mortality exceeding 90% due to ingestion and/or topical application (Fig. 2). These values were significantly higher than those obtained with limonene, the EOs of *B. anomala, B. milleflora B. trimera*, and *B. uncinella*, or azadirachtin-based bioinsecticide (topical application [F = 212.32; d.f. = 9, 45; *P* < 0.0001)]; ingestion [F = 194.3; d.f. = 9, 36; *P* < 0.0001]), which caused mortality rates of between 65 and 81% in the ingestion and topical application bioassays (Fig. 2). All of the products tested resulted in significantly (*P* < 0.0001) higher levels of mortality than the untreated controls (Fig. 2).

Based on the concentration–response curves and the overlapping confidence intervals of the LC_50_ and LC_90_ values for the ingestion and topical application bioassays, we found that these values were lower for all *Baccharis* EOs and limonene than for the spinosyn- and azadirachtin-based insecticides after 120 h of exposure (Table 2). Topical application of the *Baccharis* EOs and spinosyn showed no difference in LT_50_ values, which ranged from 4.55 to 8.71 h (Table 3). Meanwhile, the spinosyn-based insecticide had the lowest LT_50_ value in the ingestion bioassay (17.95 h; Table 3).

**Table 2:** Estimation of LC_50_ and LC_90_ (in mg L^−1^) and confidence interval of *Baccharis* spp., limonene, spinosyn-based synthetic insecticide and azadirachtin on adults of *Drosophila suzukii* at 120 HAE in topical bioassays and ingestion.

Treatments	*Slope* ± SE	LC_50_ (95% CI)^a,b^	LC_90_ (95% CI)^a,b^	χ^2c^	df
**Ingestion bioassay**					
*Limonene*	2.90 ± 0.34	19.81 (18.54–22.15) b	25.11 (23.11–26.14) b	9.08	6
*B. anomala*	2.81 ± 0.42	11.64 (8.74–13.45) a	18.98 (17.10–20.05) a	8.13	6
*B. calvescens*	3.12 ± 0.31	8.89 (6.83–10.45) a	17.42 (16.08–20.15) a	7.12	6
*B. mesoneura*	2.98 ± 0.24	6.71 (5.12–9.11) a	17.02 (16.04–19.78) a	8.11	6
*B. milleflora*	2.64 ± 0.32	6.44 (5.74–9.15) a	19.23 (18.78–20.07) a	8.45	6
*B. oblongifolia*	3.10 ± 0.43	6.52 (5.02–9.74) a	18.13 (16.17–20.19) a	7.12	6
*B. trimera*	2.67 ± 0.30	10.42 (8.16–11.11) a	21.04 (17.78–22.04) a	9.75	6
*B. uncinella*	3.14 ± 0.27	7.82 (6.57–10.14) a	17.20 (16.01–18.56) a	8.19	6
Spinetoram	2.79 ± 0.21	25.40 (21.50–27.17) b	51.89 (48.6–53.17) c	9.76	6
Azadirachtin	2.78 ± 0.23	160.14 (155.1–162.45) c	310.45 (304.4 ± 318.03) d	8.12	6
**Topical application bioassay**					
*Limonene*	2.14 ± 0.13	11.52 (9.10–13.12) b	29.74 (27.11–30.05) b	7.13	6
*B. anomala*	3.11 ± 0.42	5.94 (3.72–7.10) a	18.74 (16.11–20.15) a	9.12	6
*B. calvescens*	2.75 ± 0.53	3.40 (2.75–5.10) a	19.45 (17.83–21.14) a	6.04	6
*B. mesoneura*	2.89 ± 0.42	4.14 (3.74–5.01) a	18.11 (17.54–20.04) a	8.13	6
*B. milleflora*	3.12 ± 0.32	5.69 (3.15–7.97) a	19.75 (17.45–20.12) a	8.02	6
*B. oblongifolia*	2.98 ± 0.54	3.12 (2.66–3.89) a	22.15 (16.54–25.19) a	7.74	6
*B. trimera*	3.10 ± 0.64	5.83 (3.78–5.19) a	16.11 (13.07–22.78) a	6.82	6
*B. uncinella*	2.96 ± 0.89	7.76 (4.40–8.75) a	21.07 (16.01–23.98) a	7.02	6
Spinetoram	3.75 ± 0.11	10.55 (10.05–12.11) b	54.13 (49.54–58.11) c	8.10	6
Azadirachtin	2.18 ± 0.10	204.13 (199.66–206.11) c	416.84 (399.14–420.16) d	8.25	6

*df* degrees of freedom.

^a^LC_50_ and LC_90_: Insecticide concentrations (mg L^−1^) required to kill 50% or 90% of D. suzukii adults, respectively (CI 95% confidence interval).

^b^LC_50_ and LC_90_ values designated by different letters within a column are significantly different from each other through nonoverlap of 95% Cis.

^c^P > 0.05 in the goodness-of-fit test.

**Table 3:** Estimation of the median lethal time (LT_50_, in h) and confidence interval of formulations with *Baccharis* spp*.*, limonene, spinosyn-based synthetic insecticide and azadirachtin on *Drosophila suzukii* adults using the maximum concentration tested.

Treatments	Concentration (mg L^−1^)	Slope ± SE	LT_50_ (95% CI)^a,b^	χ^2^^c^	df
**Ingestion bioassay**					
*Limonene*	80	2.95 ± 0.74	66.41 (50.10–70.32) b	4.12	27
*B. anomala*	80	2.89 ± 0.16	43.24 (40.11–50.12) b	7.11	27
*B. calvescens*	80	3.87 ± 0.15	48.05 (41.18–55.19) b	8.15	27
*B. mesoneura*	80	3.14 ± 0.21	43.16 (39.17–50.12) b	9.20	27
*B. milleflora*	80	3.00 ± 0.17	46.30 (40.14–53.12) b	7.14	27
*B. oblongifolia*	80	2.98 ± 0.23	55.42 (41.19–60.02) b	8.11	27
*B. trimera*	80	2.87 ± 0.10	42.76 (37.13–50.12) b	9.76	27
*B. uncinella*	80	2.99 ± 0.45	52.48 (42.19–59.13) b	8.30	27
Spinetoram	75	3.09 ± 0.41	17.95 (11.12–24.98) a	9.75	27
Azadirachtin	250	2.72 ± 0.22	60.10 (50.07–69.43) b	7.12	27
**Topical Application Bioassay**					
Limonene	80	2.98 ± 0.45	11.78 (10.12–13.20) b	8.97	27
*B. anomala*	80	3.07 ± 0.31	7.76 (5.15–9.72) a	7.11	27
*B. calvescens*	80	2.17 ± 0.24	8.71 (6.45–9.32) a	5.23	27
*B. mesoneura*	80	2.15 ± 0.32	4.89 (3.73–7.11) a	6.10	27
*B. milleflora*	80	3.83 ± 0.43	4.76 (2.75–6.89) a	7.14	27
*B. oblongifolia*	80	3.67 ± 0.30	4.55 (4.00–6.45) a	8.12	27
*B. trimera*	80	3.94 ± 0.27	4.96 (3.42–5.12) a	7.94	27
*B. uncinella*	80	2.97 ± 0.31	7.10 (5.12–9.25) a	5.17	27
Spinetoram	75	3.11 ± 0.23	6.04 (4.13–8.19) a	9.32	27
Azadirachtin	250	3.80 ± 0.34	20.69 (19.13–22.74) c	9.45	27

*df* degrees of freedom.

^a^LT_50_: time required to kill 50% of D. suzukii adults following exposure to treatments (CI 95% confidence interval).

^b^LT_50_ values designated by different letters within a column are significantly different from each other through nonoverlap of 95% Cis.

^c^P > 0.05 in the goodness-of-fit test.

**Figure 3:**
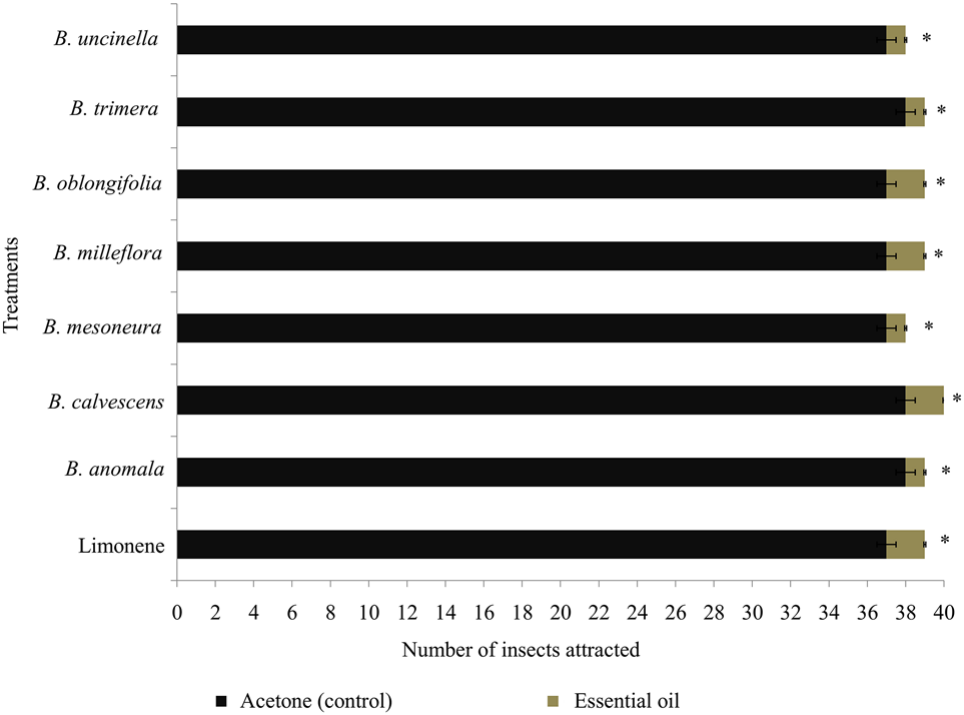
Repellence of *Drosophila suzukii* adults in the in bioassays with two-way olfactometer. Asterisks indicate significant differences between treatments according to Student’s t-test (*P* < 0.05).

**Figure 4:**
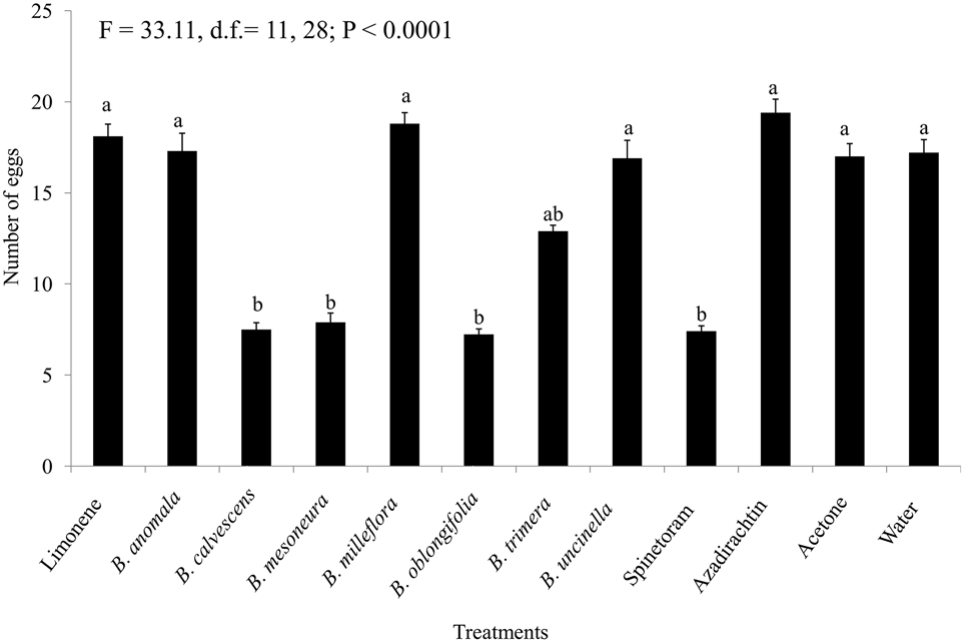
Number of eggs of *Drosophila suzukii* in artificial fruits following immersion in treatments. Bars (± SE) with the same letter are not significantly different (GLM with a quasi-binomial distribution 474 followed by Tukey post hoc test: *P* < 0.05).

When the repellent action of *D. suzukii* females was evaluated using olfactometers, it was observed that 92% of insects were repelled by treatments containing EOs, and 8% were repelled by the solvent (acetone; Fig. 3). In addition, the dry residues of the EOs significantly reduced (F = 33.11; d.f.: 11, 28; *P* < 0.0001) oviposition by *D. suzukii* on artificial fruits treated with *B. calvescens* (7.5 eggs fruit^−1^), *B. mesoneura* (7.9 eggs fruit^−1^), and *B. oblongifolia* (7.2 eggs fruit^−1^) when compared to negative controls with water (17.2 eggs fruit^−1^) and acetone (17.6 eggs fruit^−1^) (Fig. 4).

**Figure 5:**
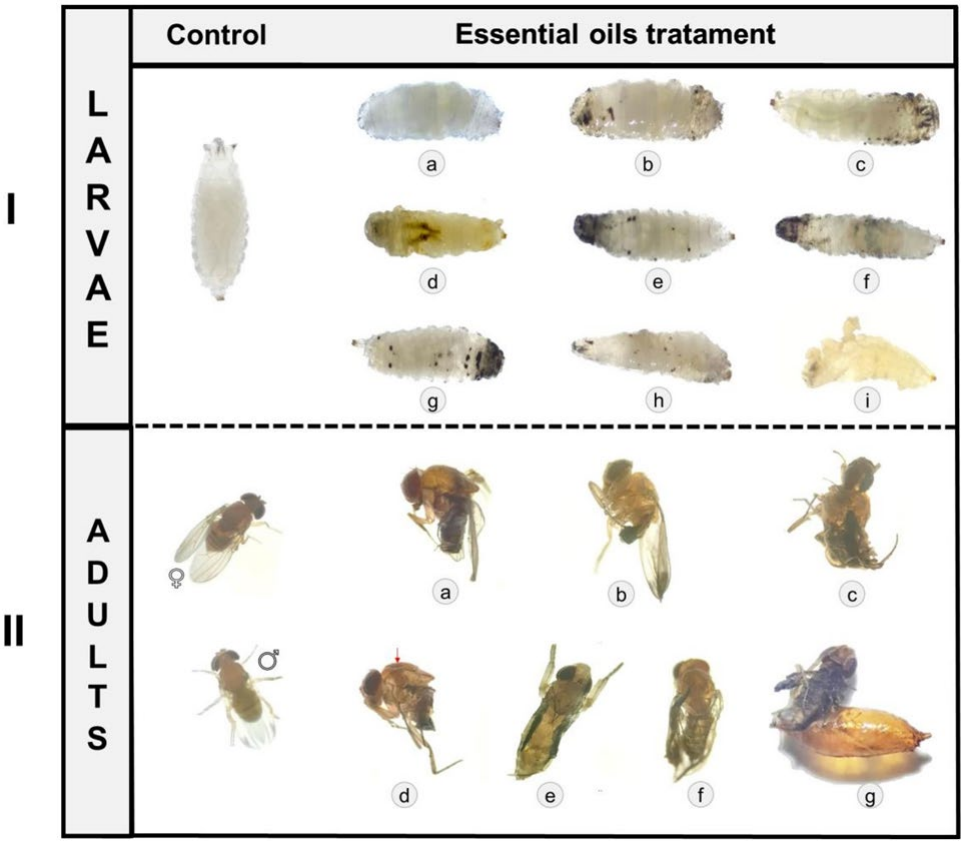
Macroscopic damage to larvae and adults of *Drosophila suzukii* after treatment with essential oils (EOs) from leaves of *Baccharis* spp*.* and limonene (40×). (**I,II**) No color change or deformity was observed in *D. suzukii* larvae and adults 2 h after the treatments (control group). (**I**) (a,b) swelling of L3 epithelial cells treated with limonene and *Baccharis trimera*, respectively. (**I**) (c,d) darkening in the respiratory filaments of L3 treated with *B. calvescens* and *Baccharis oblongifolia*, respectively. (**I**) (e–h) diffuse pigmentation in the cuticle of L3 treated with *B. anomala*, *B. mesoneura*, *B. milleflora*, and *B. uncinella*. (**I**) (h,i) deformations and skin flakes of L3 treated with *B. calvescens* and *B. oblongifolia*. (**II**) (a,b) emergence and incomplete development following treatment with *B. anomala* and limonene. (**II**) (c–g) deformities in the abdomen and wings treated with *B. mesoneura*, *B. milleflora*, *B. oblongifolia*, *B. uncinella*, and limonene. (**II**) (c–e); (g) leg deformities treated with *B. anomala*, *B. mesoneura*, *B. uncinella*, and limonene. (**I**) (d) deformities in the pronotum treated with *B. oblongifolia*. All larvae and adults were assessed at a discriminatory concentration of 8% of EOs.

All *Baccharis* spp. EOs and limonene caused greater larval mortality than controls with water or acetone (F = 22.14; d.f. = 9, 95; *P* < 0.0001), especially *B. anomala*, *B. calvescens*, *B. mesoneura*, *B. milleflora*, and *B. oblongifolia*, which caused larval mortality of ≅ 94% (Table 4). A similar effect was also observed in the biological parameters of the pupation rate (F = 36.11; d.f. = 8, 95; *P* < 0.0001) and pupal mortality (F = 17.10; d.f. = 8, 95; *P* < 0.0001; Table 4). Also, these EOs and limonene caused macroscopic abnormalities on the surface of the cuticles of larvae, including diffuse pigmentation (Fig. 5Ib–f,h), darkening of the respiratory filaments (Fig. 5If,g), deformations and flaking (Fig. 5Ih,i), as well as decreased motility in *D. suzukii* L3 following a 2 h exposure to the treatments. Adult abnormalities were also observed, such as incomplete development (Figs. 4IIa, 5IIb), deformities in the abdomen (Fig. 5IIc–g), wings (Fig. 5IIc–g), legs (Fig. 5IIc,e), and pronotum (Fig. 5IIe). These effects were not observed in *D. suzukii* larvae and adults in treatments containing only water or acetone (Fig. 5).

**Table 4:** Larval mortality (LM), pupation rate (PR), pupal mortality (PM), and deformity of *Drosophila suzukii* adults exposed to different treatments.

Treatments	LM (%)	PR (%)	PM (%)
*Limonene*	88.0 ± 4.06 ab	12.0 ± 4.06 b	95.3 ± 4.70 b
*B. anomala*	94.0 ± 1.87 a	6.0 ± 1.65 bc	100.0 ± 0.00 b
*B. calvescens*	97.0 ± 1.22 a	3.0 ± 1.22 c	100.0 ± 0.00 b
*B. mesoneura*	99.1 ± 0.99 a	1.0 ± 0.97 c	100.0 ± 0.00 b
*B. milleflora*	95.0 ± 2.73 a	5.0 ± 2.7 c	100.0 ± 0.00 b
*B. oblongifolia*	100.0 ± 0.00 a	–	–
*B. trimera*	86.0 ± 3.67 b	14.0 ± 3.67 b	96.3 ± 3.70 b
*B. uncinella*	85.0 ± 4.74 b	15.0 ± 4.74 b	100.0 ± 0.00 b
Acetone	0.00 ± 0.00 c	100.0 ± 0.00 a	0.0 ± 0.00 a
Water	0.00 ± 0.00 c	100.0 ± 0.00 a	0.0 ± 0.00 a
F	22.14	36.11	17.10
d.f.	9, 95	8, 95	8, 95
*P* values	< 0.0001	< 0.0001	< 0.0001

Columns followed by the same letter are not significantly different from one another (GLM with an almost binomial distribution followed by Tukey’s test: *P* > 0.05).

**Figure 6:**
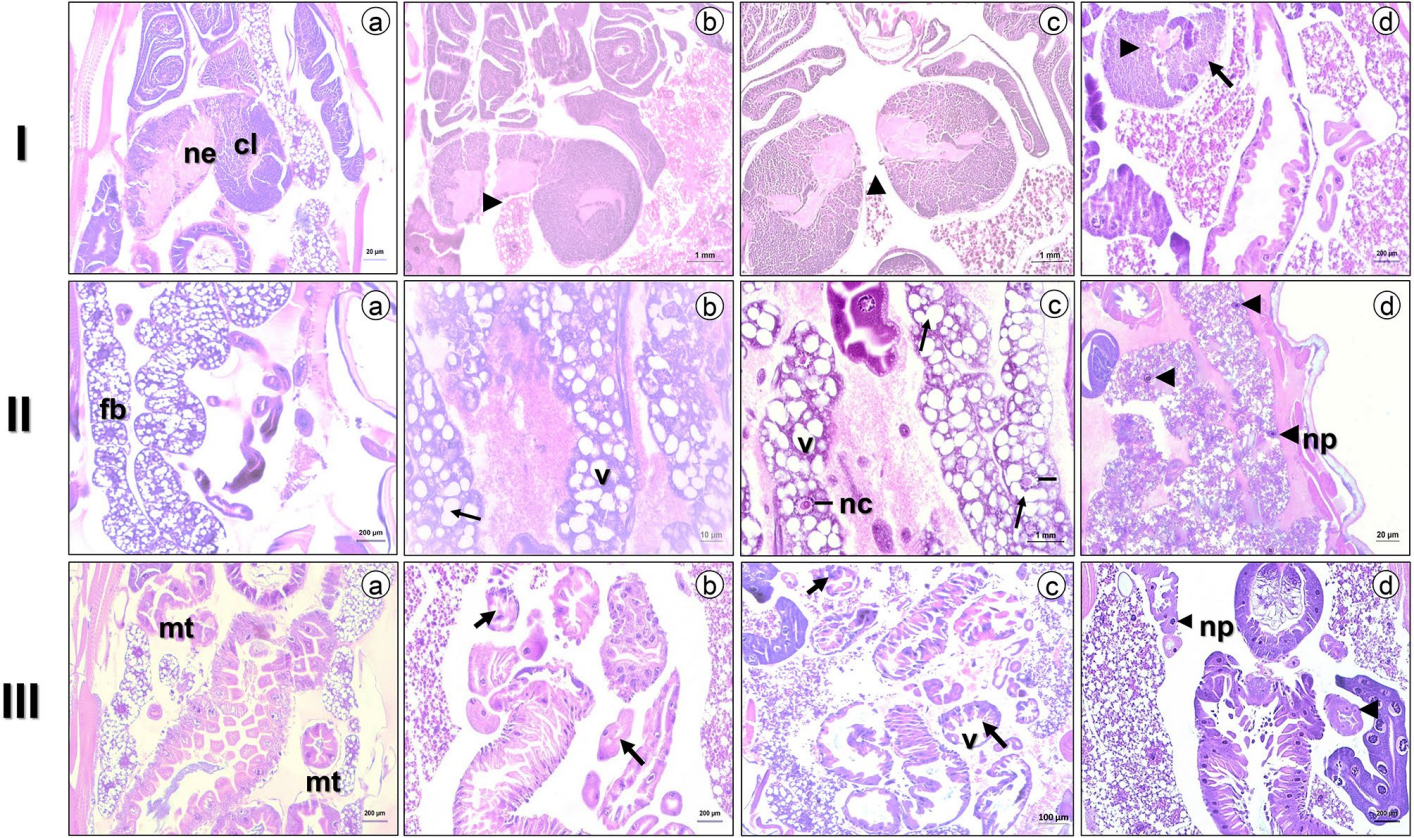
Photomicrographs of the brain ((**I**) a), fat body ((**II**) b), and Malpighian tubules ((**III**) a) of *Drosophila suzukii* L3. (a) Normal control groups 2 h after treatment (only acetone) (20 x). (**I**) (a) Observe the normal neuropils (ne) and cortical layers (cl). (**I**) (b–d) The brain of *D. suzukii* L3, 2 h after treatment with *B. calvescens*, *B. mesoneura*, and *B. oblongifolia*, respectively, with neurodegeneration and alteration of the morphology of the cortical layer (arrows) and neuropils (arrowheads). (**II**) (b,c) Observe the irregular trophocytes of the adipose body (arrows), intense cytoplasmic vacuolization (V), and condensed nuclear chromatin (-nc) after treatment with *B. anomala* and *B. mesoneura*, respectively. (**II**) (d) Note that the nuclei of the fatty body of *D. suzukii* larvae were divided into smaller fragments with the presence of nuclear chromatin and pyknotic nuclei (np) when exposed to the constituent limonene (arrowheads). (**III**) (b–d) Details of the brush border disintegration (arrows), intense cytoplasmic vacuolization (V), and condensation of nuclear chromatin from Malpighian tubules (arrowhead) observed for *B. milleflora*, *B. trimera*, and *B. uncinella*, respectively. All larvae were evaluated at the 8% discriminatory concentration of the EOs.

Untreated *D. suzukii* larvae showed histological sections with well-defined morphology of the nervous system (Fig. 6Ia), the fat body (Fig. 6IIa), and the Malpighian tubules (Fig. 6IIIa). Larvae treated with *Baccharis* EOs and limonene exhibited intense degeneration in the nervous system and the area of the neuropil (arrowheads; Fig. 6Ib–d), as well as irregular morphology of the cortical layer of the brain (arrow; Fig. 6Id). The fat body cells showed trophocytes with irregular morphology (arrows; Fig. 6IIb,c), changes related to nuclear chromatin condensation (dashed line; Fig. 6IIc), intense cytoplasmic vacuolization, and pycnotic nuclei (arrowhead; Fig. 6IId). The Malpighian tubules showed disintegration of the brush border (arrows; Fig. 6IIIb,c), intense vacuolization, and nuclear chromatin condensation (pyknotic nuclei; arrowheads; Fig. 6IIId).

## Discussion

This study provides the first verification that EOs extracted by hydrodistillation from the leaves of seven species of *Baccharis* and limonene, a constituent of these EOs, exhibit high toxicity against adults and larvae of *D. suzukii*. The EOs of *Baccharis* species are known for the predominance of monoterpenoids and sesquiterpenoids^1,4,5^, which have been reported to have the potential to cause mortality in different larval stages^31^, malformations in adults^31^, and to repel insects^7^. Of the different *Baccharis* species examined in this study, the only one whose oil has been reported in the literature as having insecticidal properties is *B. trimera*, which has been shown to be effective against pests of stored products^32^.

The gas chromatography-mass spectrometry (GC–MS) analysis showed that limonene was the only major constituent found in all *Baccharis species*, the content of which varied between 12.5 and 88.8% in the studied species. In Brazil, limonene is a product marketed for use in treatments against fleas in domestic animals in the form of shampoos, sprays, and aerosols^8^. However, previous studies have found that the compound exhibits toxic activity against several arthropods, such as *Leptinotarsa decemlineata* Say (Coleoptera: Chrysomelidae^)^^33^, *Sitophilus zeamais* Motschulsky, (Coleoptera: Curculionidae)^34^, *Tribolium confusum* du Val (Coleoptera: Tenebrionidae)^35^, *Tribolium castaneum* Herbst (Coleoptera: Tenebrionidae)^34^, and *Tyrophagus putrescentiae* Schrank (Acari: Acaridae)^36^. Besides, in vitro bioassays reduced feeding by larvae of *Thaumetopoea pityocampa* Schiff (Lepidoptera: Thaumetopoeidae) on leaves of *Pinus* spp.^37^ and feeding by *Bemisia tabaci* Gennadius (Hemiptera: Aleyrodidae) on *Solanum esculentum* L.^38^; they also had a repellent effect on *Coptotermes formosanus* Shirak (Isoptera: Rhinotermitidae) in wood^39^. Similar effects were observed in the larvae and adults of *D. suzukii* in this study. We also found high concentrations of other constituents, including α-pinene, β-pinene, spatulenol, and thujopsan-2-α-ol (*B*. *calvescens*); carquejyl acetate and palustrol (*B. trimera*); β-pinene*(B. milleflora*); α-pinene, β-pinene (*B. mesoneura*); α-thujene, α-pinene, β-pinene (*B. oblongifolia*); and β-pinene, thujopsan-2-α-ol, globulol (*B. anomala*). These findings are corroborated by previous studies that found that *Baccharis* species contained large amounts of monoterpene hydrocarbons (α-thujene, α-pinene, β-pinene, and limonene), oxygenated monoterpenes (carquejyl acetate), and oxygenated sesquiterpenes (palustrol, spathulenol, and thujopsan-2-α-ol)^5^.

However, for both bioassays performed in this study, we found that the substances contained in the EOs of *B. calvescens*, *B. mesoneura*, and *B. oblongifolia* had the greatest effect on adults of *D*. *suzukii*, with similar mortality rates (over 90%) to synthetic spinosyn-based insecticides. These species showed efficacy comparable to the organophosphates, pyrethroids and spinosyns used to management adults of *D. suzukii*^40–43^. It is known that the potential of EOs depends on the chemical constituents and their proportions found in the samples^44^. Likewise, the interactions of constituents contained in EOs have been reported to have synergistic action, providing a significant increase in the effectiveness of formulations^44,45^. The insecticide azadirachtin, meanwhile, showed the lowest toxicity on adults of *D. suzukii*. However, even though this product exhibits low toxicity for this pest, it may favor pest suppression by repelling the insects or reducing oviposition capacity, as verified in a previous study^40^.

In the topical application bioassays, we observed that adults of *D. suzukii* died more quickly (LT_50_ of 4.55–11.78 h) than during the ingestion bioassays (LT_50_ of 42.76–66.41 h). This difference in the toxicity of *Baccharis* spp. oils evaluated using the two bioassay methods can be attributed to the fact that topically applied EOs directly penetrate the insect hemolymph in a single dose. In contrast, ingested EOs are administered gradually and in small amounts over the feeding period (24 h). This also suggests that the higher toxicity by topical application results from damage to the nervous and/or respiratory systems of insects since these are the main routes of intoxication by substances absorbed by the cuticle^46^. Furthermore, during the ingestion period, treatments remain in the intestine of the insects for longer, requiring a longer time for metabolization and/or excretion of the chemical^29^. These results too may be related to the lipophilic constitution and the low molecular weight of the chemical constituents of these EOs^47^. These characteristics may enable diffusion through the cellular membrane, causing physiological disruptions in the insect membrane and leading to mortality^48,49^. Likewise, they can trigger the inhibition of acetylcholinesterase (AChE) activity, which has been verified in adults of *S. zeamais*, *T. castaneum*^50^ and the spider mite, *Tetranychus urticae* Koch (Acari^)^^51^.

In addition to their high toxicity, the EOs of *B. calvescens*, *B. mesoneura*, and *B. oblongifolia* reduced the oviposition capacity of *D. suzukii* by up to 43%. This fact corroborates the observations in the double-choice olfactometry repellency tests, in which females of *D. suzukii* avoided the olfactometer arm that contained a piece of filter paper containing 5 µL of EOs, preferring instead to move into arm containing the negative treatment (acetone). Products that reduce oviposition or repel *D. suzukii* females can reduce the incidence of epidermal rupture by oviposition, which consequently reduces phytopathogen infestation^43^, while also avoiding the attraction of other drosophilids such as *Zaprionus indianus* Gupta (Diptera: Drosophilidae), which can accelerate damage to crops, as seen in strawberry^13^ and persimmons^15^. In addition, it helps decrease pest population density in crops^13,29^.

In addition to their repellent effects and, consequently, their ability to reduce oviposition, *B. anomala*, *B. calvescens*, *B. mesoneura*, *B. milleflora*, *B. oblongifolia*, and limonene had a major impact on the L3 larvae of *D. suzukii*. Specifically, they were able to affect the species’ pupation rate and pupal mortality negatively. The larvicidal effect of these materials may be related to the polarity of the EOs (lipophilic substances), which allows oils to penetrate the cuticle of the larvae, interfering in their physiological functions^52^ and directly hindering their development^52,53^. Other authors investigating sublethal effects of sub-lethal doses (LD_30_ = 25 mg/L) of *Cinnamomum verum* J. Presl EO administered to 4th instar larvae of *Culex quinquefasciatus* Say (Diptera: Culicidae) observed reduced adult emergence, decreased fertility (eggs/females) and egg fertility^54^. Likewise, using *Carlina acaulis* L. EO (LD_30_ = 3.9 µg fly^−1^) against *Musca domestica* L. (Diptera: Muscidae) where there was a negative impact on females’ fertility, as well as, emerged larvae have less vitality as a result of insufficient food intake and higher mortality during the juvenile phase^55^. Besides, *M. domestica* adult exposure to *Thymus vulgaris* L. EO (LD_30_ = 25.5 µg fly^−1^) reduced adult longevity, F1 vitality and F1 fecundity^56^.

Most insecticides used to control *D. suzukii* act on the AChE receptors or the sodium^23–25,41,57,58^. Products that use a different mode of action can thus help avoid the emergence of resistance to such compounds^27,28^. Studies of these morphological markers, including the histopathological evaluation of larvae, is of the utmost importance when seeking to understand how exposure to EOs and their individual constituents can damage target cells. In this study, we observed morphological damage to organs such as the brain, fat body, and Malpighian tubules of *D. suzukii* larvae subjected to the EOs of *Baccharis* spp*.* and limonene. We examined these organs in particular because the brain is the organ that transmits the stimuli received through physical and chemical impulses, while the fat body and Malpighian tubules are the main sites of metabolization and excretion of substances, analogous to the liver and kidney of vertebrates, respectively^59^.

In this study, *B. calvescens*, *B. mesoneura*, and *B. oblongifolia* were shown to have neurotoxic mechanisms, including the neurodegeneration and alteration of the morphology of the cortical layer and neuropils. Similar observations were reported with larvae of *Cochliomyia macellaria* (Diptera: Calliphoridae) after being treated with the oil of *Curcuma longa* L.^52^. In that study, the authors demonstrated the occurrence of vacuolar degeneration and alteration of the hypnotic profile of the brain. Also, *D. suzukii* larvae exposed to EOs showed damage to the adipose body, including cytoplasmic vacuolization and irregular morphology of trophocytes with hypnotic nuclei, signaling a possible mechanism of excretion of EOs. This process of vacuolization may indicate that these cells are in the process of dying, as has been demonstrated in larvae of *C. quinquefasciatus*^60^. Besides, we observed that the fat body nucleus of *D. suzukii* larvae was divided into smaller, highly condensed fragments when exposed to limonene and advanced disintegration of the brush border and nuclear chromatin condensation of the Malpighian tubules was caused by the EOs of *B. milleflora*, *B. trimera*, and *B. uncinella*. These results corroborate those described for *Apis mellifera* (Hymenoptera: Apidae^)^^61^ and *C. macellaria*^52^. These physiological disturbances caused by EOs and limonene in *D. suzukii* larvae are typical of cells submitted to classical apoptosis^62^, consisting of self-destruction of cells into smaller, highly condensed fragments.

The results found in the study of larval and adult *D. suzukii* clearly demonstrate the toxic activity and sublethal effects of the EOs of *Baccharis* spp*.* and limonene, an isolate of these EOs. Furthermore, this study is the first to verify the histological effects of EOs on *D. suzukii* larvae. This can help to determine the action sites of these compounds on insects. However, considering that these findings has not been fully explained, we are aware that new tests, focused especially on the selectivity of these botanists over natural enemies intentionally released^63,64^ and naturally present in the environment^65,66^, may in the future subsidize methods for integrating natural enemies and the development of EO-based biopesticides. Despite this, the use of these substances as such has limitations due to flammability, low dispersion in water, phytotoxicity^67–69^. In this sense, the development of formulations based on stable EO reduces these negative aspects and, at the same time, improves the effectiveness against pests and reduces the side effects on the beneficial ones. We are currently conducting work to investigate the domestication of the species and the optimization of EO extraction processes, as well as to determine how to stabilize the active components in formulations based on micro and nanoencapsulation.

## Material and methods

### Collection of plant material for essential oil extraction

Table 5 summarizes information on the selected species of *Baccharis* (*B. anomala*, *B. calvescens*, *B. mesoneura*, *B. milleflora*, *B. oblongifolia*, *B. trimera*, and *B. uncinella*) used in the treatments and control. The species were identified by the specialist Osmar dos Santos Ribas and vouchers were deposited at the Municipal Botanical Museum (MBM Herbarium) in Curitiba, Paraná, Brazil (25° 28′ 37.90 S, 49° 59′ 34.50 W and 960 m altitude). The collected leaves were cut into segments of approximately 2 cm, and EOs were hydrodistilled in a Clevenger-type apparatus (Vidrolabor, São Paulo, Brazil) for 4 h and 30 min. Subsequently, the hydrolate was separated using anhydrous sodium sulfate. The samples were kept in a freezer at − 20 °C until chemical analysis was carried out. We decided to include limonene on its own in the bioassays because it is the only major constituent (≥ 10%) present in all species studied. Samples of D-limonene (CAS: 5989-27-5) were obtained from Sigma-Aldrich Brazil (São Paulo, Brazil) with ≥ 99% purity.

**Table 5:** Insecticides evaluated for the management of *Drosophila suzukii.*

Treatments	Description^a^	Geographic coordinates of origin	Discriminatory concentration tested^b^	Origin/manufacturer
Limonene EO			80	Sigma Aldrich (São Paulo, SP, Brazil)
*Baccharis anomala* EO	Essential oil extracted from the leaves of *Baccharis anomala* DC. (pre-commercial)	25° 29′ 45.04′′ S 48°59′ 56.58′′ W	80	Laboratory extraction and formulation (Curitiba, Paraná, Brazil)
*Baccharis calvescens* EO	Essential oil extracted from the leaves of *Baccharis calvescens* DC. (pre-commercial)	25° 30′ 18.44′′ S 49° 1′ 14.47′′ W	80	Laboratory extraction and formulation (Curitiba, Paraná, Brazil)
*Baccharis mesoneura* EO	Essential oil extracted from the leaves of *Baccharis mesoneura* DC. (pre-commercial)	25° 29′ 33.95′′ S 49° 0′ 41.05′′ W	80	Laboratory extraction and formulation (Curitiba, Paraná, Brazil)
*Baccharis milleflora* EO	Essential oil extracted from the leaves of *Baccharis milleflora* DC*.* (pre-commercial)	25° 30′ 38.67′′ S 49° 0′ 24.12′′ W	80	Laboratory extraction and formulation (Curitiba, Paraná, Brazil)
*Baccharis oblongifolia* EO	Essential oil extracted from the leaves of *Baccharis oblongifolia* Pers. (pre-commercial)	25° 30′ 38.67′′ S 49° 0′ 51.23′′ W	80	Laboratory extraction and formulation (Curitiba, Paraná, Brazil)
*Baccharis trimera* EO	Essential oil extracted from the leaves of *Baccharis trimera* (Less) DC. (pre-commercial)	25° 28′ 37.90′′ S 48° 59′ 34.50′′ W	80	Laboratory extraction and formulation (Curitiba, Paraná, Brazil)
*Baccharis uncinella* EO	Essential oil extracted from the leaves of *Baccharis uncinella* DC. (pre-commercial)	25° 31′ 4.29′′ S 48° 59′ 57.55′′ W	80	Laboratory extraction and formulation (Curitiba, Paraná, Brazil)
Delegate	Spinetoram (250 g kg^−1^)		75	Corteva Agriscience (São Paulo, São Paulo, Brazil)
Azamax	Azadirachtin (12 g L^−1^)		250	UPL Brazil, Ltda (Campinas, São Paulo, Brazil)

^a^Laboratory of Ecophysiology, Federal University of Paraná (Extraction) and Laboratory of Semiochemistry, Federal University of Paraná (Formulation), Curitiba, Paraná State, Brazil.

^b^Concentration: 75 mg of commercial product per L of water (Delegate); 250 mL of commercial product per 100 L of water (Azamax); 80 mg L^−1^ (0.16 µL) of essential oils per 2 mL of acetone.

### Chemical analysis of essential oils: identification and quantification

We performed GC–MS using a Shimadzu 2030 gas chromatograph coupled to a Shimadzu TQ8040 sequential mass detector (GC–MS/MS). The GC was equipped with a fused HP-5MS capillary column (film thickness 30 m × 0.25 mm × 0.25 μm) coated with a stationary phase of 5% phenyl-95% dimethylpolysiloxane. Helium was used as a drag gas at a flow rate of 1.0 mL min^−1^. The temperature setting was set to increase from 60 to 240 °C at a rate of 3 °C min^−1^ and held at 240 °C for 10 min. The injector temperature was maintained at 250 °C. The essential oil samples were diluted into a 1% hexane solution, and 1.0 μL of the solution was injected with a partition ratio of 1:30. The mass detector was operated in electron impact mode (70 eV). The transfer line was kept at 260 °C and the ion source at 250 °C. For quantification, essential oils were injected, and the Shimadzu GC 2030, equipped with a flame ionization detector (FID), was operated at 250 °C. Synthetic air was used as a carrier gas at a flow rate of 1.5 mL min^−1^, using the same column and conditions described above. The quantification of each constituent was estimated by the FID detector with the corresponding peak area, which was determined using the average of three injections (Table 1). The identification of the SB components was performed by comparing the mass spectra with those of commercial libraries^70^, as well as by their linear retention rates^71^, after the injection of a homologous series of alkanes (C_8_–C_26_) under the same experimental conditions, and compared with data in the literature^72^. The structure of limonene was confirmed by injecting a commercial standard solution (Sigma-Aldrich Brazil).

### Breeding and maintenance of *Drosophila suzukii*

The adults of *D. suzukii* used in bioassays were in their tenth generation. Breeding was performed using insects collected in the strawberry fields *(Fragaria* × *ananassa* Duchesne) in January 2018 in Curitiba, Paraná, Brazil (31° 38′ 20′′ S, 52° 30′ 43′′ W). In the laboratory, the infested strawberries were placed individually in plastic jars (150 mL) with a perforated lid (2 cm in diameter) and covered with cheesecloth containing a thin layer of vermiculite (1 cm). The fruits were kept in an air-conditioned room (25 ± 2 °C, 70 ± 10% RH, and 12-h photoperiod) until the emergence of adults. Following emergence, the adults (males and females) were transferred to glass bottles (300 mL) containing an artificial diet (12 mL)^73^. Seven-day-old adults were used in all bioassays, which were deprived of food for 8 h, though they were supplied with water in hydrophilic cotton.

### Bioassays

All bioassays were conducted under controlled conditions (25 ± 2 °C, 70 ± 10% RH, and 12-h photoperiod) using a completely randomized design. The treatments and discriminatory concentrations used are listed in Table 5. Concentrations (solutions) of 2.5, 5.0, 7.5, 10, 20, 40, and 80 mg L^−1^ of the intact EOs of *Baccharis spp.* (*B. anomala*, *B. calvescens*, *B. mesoneura*, *B. milleflora*, *B. oblongifolia*, *B. trimera*, and *B. uncinella*) and limonene were prepared by diluting all treatments in acetone (PanReac-UV-IR-HPLC-GPC PAI-ACS, 99.9% purity). A spinosyn-based insecticide (Spinetoram–7.5 mg a.i. L^−1^; Delegate 250WG, Dow AgroSciences, Santo Amaro, São Paulo, Brazil) and an azadirachtin-based bioinsecticide (azadirachtin+3-tigloyl-azadiractol, 1.2 mL a.i. L^−1^; Azamax 1.2 EC, UPL Brazil, Campinas, São Paulo, Brazil) were used as positive controls (Table 5). The solvent (water or acetone) used in the solubilization of the respective treatments were used as negative controls.

### Discriminatory bioassays (initial experiment)

In order to evaluate the lethal toxicity of *Baccharis* spp. EOs and limonene, initial tests were performed using ingestion bioassays and topical application using discriminatory concentrations on adults of *D. suzukii* (Table 5). For the ingestion bioassays, 16 adults of *D. suzukii* (eight couples) were grouped in transparent plastic cages (1 L) inverted in plastic Petri dishes (25 cm diameter). The top side of the cages (i.e., the bottom of the containers) was sealed with a cheesecloth-type fabric to allow gas exchange. Once the solutions (treatment) were prepared, the products were supplied to the flies by capillarity in hydrophilic cotton rolls inside a 10 mL glass bottle. After 24 h of exposure, the treatments were removed and replaced with an artificial diet and distilled water until the end of the evaluation period.

In the topical application bioassay, adults of *D. suzukii* (ten couples) were separated and placed in transparent glass tubes (1.3 cm in diameter × 10 cm in length), which were closed at the top with hydrophilic cotton. Subsequently, the flies were transferred to a petri dish (9 cm in diameter) lined with filter paper and sedated in ethyl ether for 40 to 60 s to apply the treatments. The solutions (2 mL) were then sprayed using a Potter Tower (Burkard Scientific, Uxbridge, UK) at a working pressure of 0.049 MPa, resulting in an average residue deposition of 1.0 mg cm^−2^. After spraying, the insects were placed in transparent plastic cages (1 L) as described above and fed an artificial diet and distilled water throughout the evaluation period.

In both tests, the experimental design was entirely randomized, with 10 treatments containing five repetitions (cages) with 16 adults (eight couples) in the ingestion bioassay and four repetitions (cages) with 20 adults (ten couples) in the topical bioassay. Mortality in each treatment was evaluated at 1 h intervals for the first 24 h after exposure to treatments (HAET) and every 24 h between 24 and 120 HAET. Insects that did not react to the touch of a fine-tip brush were considered dead. The corrected mortality was calculated using Abbott’s formula^74^.

### Concentration–response curves and average lethal time of the most promising treatments against *Drosophila suzukii*

Based on the initial bioassays, the most promising treatments were selected and submitted to a new bioassay to estimate the lethal concentrations that would result in mortality of 50% or 90% mortality among the flies (LC_50_ and LC_90_, respectively). Seven concentration ranges were defined for each treatment and exposure type in the bioassay: 25**–**80 mg L^−1^ for the EOs of *Baccharis spp.* (*B. anomala*, *B, calvescens*, *B. mesoneura*, *B. milleflora*, *B. oblongifolia*, *B. trimera*, and *B. uncinella*) and limonene; 5**–**75 mg L^−1^ for spinetoram; and 25**–**250 mg L^−1^ for commercial azadirachtin-based bioinsecticide^75^. The exposure and assessment procedures, as well as the mortality criteria, were identical to the initial tests. Four replicates were used in the ingestion bioassays, each containing 20 flies (*n* = 80) for each insecticide concentration. In the topical bioassays, five replicates were performed with 16 flies per replicate (*n* = 80) per concentration of each insecticide tested. For the determination of LT_50_ values (mean time required to kill 50% of the population) of the treatments on *D. suzukii* adults, the maximum concentration tested in the bioassays of ingestion and the topical application was used (Table 5). The experimental design and bioassay procedures were identical to those used in the initial experiments.

### Repellent effect against *Drosophila suzukii* in olfactometer bioassay

To verify the effectiveness of EOs at repelling females of *D. suzukii* relative to acetone treatments, we began by placing individual females aged up to 24 h into glass tubes (1.3 cm in diameter × 10 cm in length). In the test, the glass tube containing the female was connected to a double-choice glass olfactometer with a diameter of 8.0 cm and an initial compartment of 20 cm on each side, under fluorescent light (60 W, luminance 290 lx). At the end of one of the olfactometer arms, we placed a filter paper measuring 4 × 10 cm and bent into an accordion shape, which contained 5 µL of an EO of *Baccharis* spp. (*B. anomala*, *B. calvescens*, *B. mesoneura*, *B. milleflora*, *B. oblongifolia*, *B. trimera*, or *B. uncinella*) or limonene at the discriminatory concentration (80 mg L^−1^ of oil). Another filter paper was placed at the end of the other arm (4 × 10 cm), which contained 5 µL of acetone (control). Airflow in the system was supplied at a rate of 0.8 L min^−1^ from a previously filtered source with active carbon and humidified in distilled water. The olfactometer was washed with neutral soap and hexane after every fourth repetition and then dried in a sterilization oven at 150 °C. After this process, the substances were replaced, and the evaluation continued. Each treatment consisted of 40 replicates, each of which consisted of a female of *D. suzukii* (*n* = 40). The responses were considered positive (EOs, limonene, or acetone) when *D. suzukii* females reached the odor source or traveled at least 10 cm inside the olfactory arms and remained there for at least 1 min^53^. Flies that did not move to either of the olfactory arms after one minute of release were discarded.

### Deterrence of oviposition by *Drosophila suzukii*

Artificial fruits prepared with agar (19 g), raspberry gelatin (10 g), methylparaben (8 mL, consisting of 0.8 g dissolved in 8 mL of 99.9% ethyl alcohol; Nipagin, Vetec, Química Finz, Duque de Caxias, Rio de Janeiro, Brazil), and distilled water (reflux; 850 mL) were used as a substrate for oviposition. Using a Potter Tower (working pressure 0.049 MPa (Burkard Scientific, Uxbridge, United Kingdom) 1 mL treatments of *B. anomala*, *B. calvescens*, *B. mesoneura*, *B. milleflora*, *B. oblongifolia*, *B. trimera*, and *B. uncinella* EOs and limonene were sprayed to a mean deposition residue of 0.4 mg cm^−2^. The artificial fruits were then placed in an air-conditioned room (25 ± 2 °C, 70% ± 10% RH, and 12-h photoperiod) for three hours to let the excess moisture evaporate and, in turn, for residue deposition to occur. The fruits were placed individually in a plastic container (250 mL), covered on top with cheesecloth to allow gas exchange with the internal and external environment of the container. Five couples of *D. suzukii* (≅ 7 days old) that had previously mated were then released. After 24 h, the adults were removed, and the eggs in each fruit were counted using a Stemi 2000-C stereoscopic microscope (Carl Zeiss, Germany; × 40 magnification). The experimental design was in random blocks, with 30 replicates (fruits) per treatment.

### Lethal and sublethal effect on *Drosophila suzukii* larvae

To evaluate the larvicidal effect of *Baccharis* spp. EOs, (*B. anomala*, *B. calvescens*, *B. mesoneura*, *B. milleflora*, *B. oblongifolia*, *B. trimera*, and *B. uncinella*) and limonene, groups of 20 *D. suzukii* larvae in stage L3 were placed in transparent glass tubes (2.5 cm diameter × 8 cm length) containing a filter paper (2 × 4 cm) impregnated with 0.2 mL of EO solutions solubilized in acetone (PanReac-UV-IR-HPLC-GPC PAI-ACS, 99.9% purity). For each treatment, a discriminatory concentration of 80 mg L^−1^ of oils was used. Acetone and distilled water were used as negative control. Following EO application, the glass tubes were sealed at the top with cheesecloth to facilitate aeration and transferred to controlled conditions (25 ± 2 °C, 70 ± 10% RH, and 12-h photoperiod). The macroscopic damage to the larvae of *D. suzukii* was recorded with a Stemi 2000-C stereoscopic microscope (Carl Zeiss, Germany; × 40 magnification). The experimental design was entirely randomized with five replicates (20 larvae per replicate) for each concentration (*n* = 100). Larval mortality was assessed at 6, 24, and 48 h after the larvae and treatments were placed in the tubes. Total mortality (TM), pupation rate (PR), pupal mortality (PM), and adult deformity (AD) were calculated^53^.

### Larval histopathology of *Drosophila suzukii*

The histopathological effect of EOs on *D. suzukii* larvae was verified at a discriminatory concentration of 80 mg L^−1^ for *Baccharis spp*. EOs (*B. anomala*, *B. calvescens*, *B. mesoneura*, *B. milleflora*, *B. oblongifolia*, *B. trimera*, and *B. uncinella*) and limonene. In each treatment, groups of 20 instar III larvae (L3) were fixed in neutral buffered formalin, pH 7.2, at 10% in distilled water for 2 h at 56 °C inside microtubes (2 mL). Acetone (PanReac-UV-IR-HPLC-GPC PAI-ACS, 99.9% purity) was used as the sole negative control. After fixation, the larvae were washed three times in 70% alcohol for 20 min to remove the fixing solution. They were then dehydrated using an increasing alcoholic series (70% to 100%), remaining at each concentration for 30 min. Subsequently, the larvae were diaphanized in xylol for 10 min and transferred to soaking paraffin (overnight), and incorporated in histological paraffin. Five 4 µm thick longitudinal sections were cut with a microtome and placed on microscope slides. Mayer’s albumin was applied to the slide under the sections for bonding, after which the slide was dried at room temperature (22 ± 3 °C). Finally, the histological sections were stained with hematoxylin–eosin (H&E). Histological sections were analyzed under the Stemi 508 optical scanning microscope (Carl Zeiss, Germany; 20 or × 40 magnification), and morphological changes in target organs such as brain, fat body, and Malpighian tubules were noted.

### Statistical analysis

All bioassays were conducted using a completely randomized design. Generalized linear models (GLM)^76^ of the quasi-binomial distributions were used to analyze mortality rate data. In all cases, the fit of the GLM was determined by using the half-normal probability plot with a simulation envelope^77^. When significant differences were found among treatments, multiple comparisons (Tukey test, *P* < 0.05) via the *glht* function in the *multicomp* package with adjusted p values was performed. For comparisons of the average of two treatments in the repellency bioassay, we used the Student’s t-test. All of these analyses were carried out using R statistical software, version 2.15.1^78^. A binomial model with a complementary log–log link function (gompit model) was used to estimate the lethal concentrations (LC_50_ and LC_90_), using the Probit Procedure in the software SAS version 9.2^79^. A likelihood ratio test was used to test the hypothesis that the LCp or LTp values (lethal concentration or lethal time at which a percent mortality *P* is attained) were equal. If the hypothesis was rejected, pairwise comparisons were performed and significance was stated if CIs did not overlap. Finally, the mean lethal time (LT_50_) was estimated for Probit analysis of correlated data^80^. The percentage repellence (PR) was calculated using the formula^81^: PR (%) = [(Nc − Nt)/(Nc + Nt)] × 100, where Nc was the number of insects present in the negative control (acetone) and Nt was the number of insects present in the treatment (EOs).

## Data Availability

This article does not report new empirical data or software.
